# Profiling the transcriptomic signatures and identifying the patterns of zygotic genome activation – a comparative analysis between early porcine embryos and their counterparts in other three mammalian species

**DOI:** 10.1186/s12864-022-09015-4

**Published:** 2022-11-24

**Authors:** Yanhui Zhai, Hao Yu, Xinglan An, Zhiren Zhang, Meng Zhang, Sheng Zhang, Qi Li, Ziyi Li

**Affiliations:** 1 Key Laboratory of Organ Regeneration and Transplantation of Ministry of Education, First Hospital, Jilin University, Changchun, 130021 China; 2 College of Animal Science, Jilin University, Changchun, 130062 Jilin China; 3 Zhuhai People’s Hospital (Zhuhai hospital affiliated with Jinan University), Zhuhai, 519000 Guangdong China

**Keywords:** Transcriptomics, Zygotic genome activation, Pigs, Comparison analysis, C-MYC

## Abstract

**Background:**

The transcriptional changes around zygotic genome activation (ZGA) in preimplantation embryos are critical for studying mechanisms of embryonic developmental arrest and searching for key transcription factors. However, studies on the transcription profile of porcine ZGA are limited.

**Results:**

In this study, we performed RNA sequencing in porcine in vivo developed (IVV) and somatic cell nuclear transfer (SCNT) embryo at different stages and compared the transcriptional activity of porcine embryos with mouse, bovine and human embryos. The results showed that the transcriptome map of the early porcine embryos was significantly changed at the 4-cell stage, and 5821 differentially expressed genes (DEGs) in SCNT embryos failed to be reprogrammed or activated during ZGA, which mainly enrichment to metabolic pathways. *c-MYC* was identified as the highest expressed transcription factor during ZGA. By treating with 10,058-F4, an inhibitor of *c-MYC*, the cleavage rate (38.33 ± 3.4%) and blastocyst rate (23.33 ± 4.3%) of porcine embryos were significantly lower than those of the control group (50.82 ± 2.7% and 34.43 ± 1.9%). Cross-species analysis of transcriptome during ZGA showed that pigs and bovines had the highest similarity coefficient in biological processes. KEGG pathway analysis indicated that there were 10 co-shared pathways in the four species.

**Conclusions:**

Our results reveal that embryos with impaired developmental competence may be arrested at an early stage of development. c-MYC helps promote ZGA and preimplantation embryonic development in pigs. Pigs and bovines have the highest coefficient of similarity in biological processes during ZGA. This study provides an important reference for further studying the reprogramming regulatory mechanism of porcine embryos during ZGA.

**Supplementary Information:**

The online version contains supplementary material available at 10.1186/s12864-022-09015-4.

## Background

The early stage of mammalian embryonic development is regulated by maternal factors. Newly expressed mRNAs are continuously accumulated and updated in the embryos, thereby regulating early embryonic development [1]. In the early stages of embryonic development, the first dramatic change in gene transcription occurs during ZGA. The time point of ZGA is species-specific. It has been reported that the activation of zygotic genes in mice occurs at the 2-cell stage, while that in humans, pigs, goats and bovines occurs lightly later, at the 4-cell to 8-cell stage or even later [2–4]. Thus, each species regulates embryonic development through its own unique gene transcription patterns.

Whether the embryonic genome can initiate transcription in the early developmental stage depends on key components regulating transcription or their expression levels reaching the threshold level. The embryo needs a certain amount of time to produce these key factors, and transcriptional activation occurs once the threshold level is reached. This has been verified in the *Xenopus laevis* [5], with continuous cleavage and translation, the *TBP* transcript increases, reaching a high enough level during genome activation, and then triggers zygotic genome activation [6]. Almouzni et al. found that the lack of gene-specific TFs prior to genome activation causes transcriptional silencing [7].

*Zelda* (Zld) was the first identified zygotic genome activator in *Drosophila* [8]. *Nanog*, *Soxb1* and *Pou5f3* are important transcription factors during ZGA in zebrafish [9], which that activate the expression of hundreds of ZGA related genes. The Cairns team determined that *DUX* was the first transcription factor driving the transcription initiation of early mouse embryos [10]. It was proposed that *ZSCAN4* is a stabilizer that initiates the ZGA in placental mammals [11, 12]. *OCT4* is crucial for human major ZGA and enriched in the open chromatin region of human embryos [13, 14].

Yamanaka’s team reprogrammed terminally differentiated cells to induce pluripotent stem cells in mice through exogenous introduction of four transcription factors [15], and identified that c-*MYC* as one of the key transcription factors in the whole reprogramming process. Liu et al. found that TDG was defined as a pig-specific epigenetic regulator for nuclear reprogramming and that transient TDG overexpression promoted DNA demethylation and enhanced the blastocyst-forming rates of porcine SCNT embryos [16]. The whole genome transcript profile of pigs during maternal to zygotic transition is helpful to understand the stage-specific transcriptome, the removal of stored maternal transcripts and newly synthesized transcripts in ZGA, so as to identify key genes and signal pathways.

The efficiency of SCNT-mediated cloning in mammals, and especially including pigs, remains very low [17, 18]. For those reasons, thus far, a wide panel of investigations have been conducted to recognize, a more extensively, the biological, genetic and epigenetic factors that influence the molecular mechanisms of embryogenesis, ZGA and quality of SCNT-derived oocytes in pigs and different mammalian species [19, 20]. The source and provenance of nuclear donor cells is a fundamental prerequisite for determination of the overall outcome of propagating cloned embryos [21–23]. Incomplete ZGA has been shown to be one of the major causes affecting the development of mouse, human and porcine SCNT embryos [24–28]. Scientists have been working to find ways to improve the efficiency of cloning. Yi Zhang Laboratory and Shaorong Gao Laboratory respectively reported that histone demethylase Kdm4d could increase the cloning efficiency in mice from 1.0 to 8.7% [29–31]. Sun Qiang’s lab used the ideal combination of TSA + KDM4D to increase the cloning efficiency of macaque monkeys from 0 to 2.5% [32]. Miao Yiliang’s team and Chen Zhenxia’s team used a combination of KDM4A+ GSK126 to increase the blastocyst rate of pig cloned embryos by nearly twofold [16], but the cloning efficiency was still very low [33]. Referring to the experience of mice and other species and combining multiple high-throughput analysis methods to find the most suitable method for pigs is the direction of research to improve the efficiency of somatic cell cloning in pigs.

In our study, we revealed the transcriptional patterns of porcine IVV and SCNT embryos and comparatively analyzed the transcriptional activity of embryos in pigs, mice, bovines and humans. We further identified key genes and transcription factors during ZGA and verified the essential role of the transcription factor in porcine embryonic development, which provided an important reference for further functional investigation.

## Results

### Transcriptional activities of porcine MII oocytes and IVV embryos

**Fig. 1:**
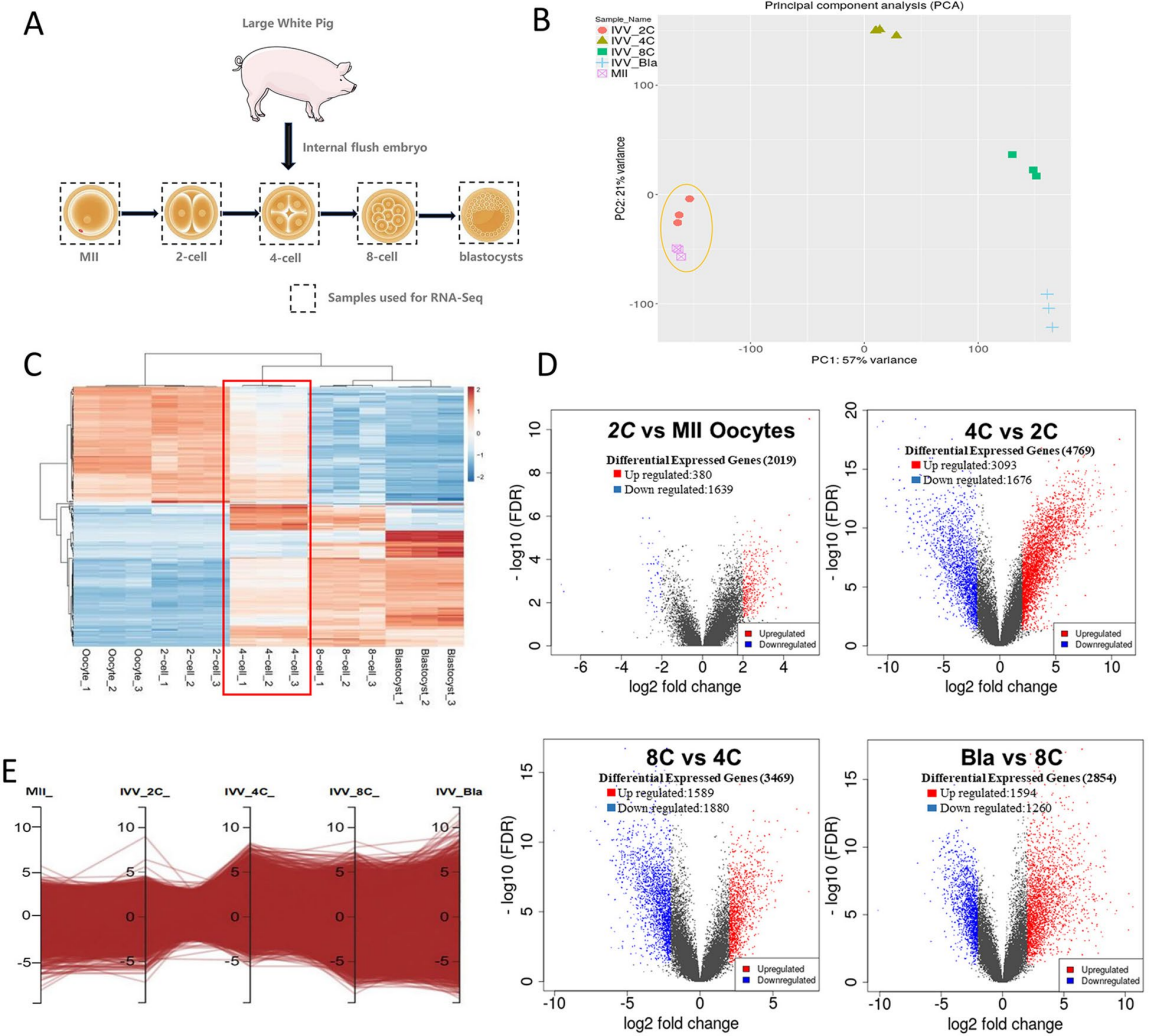
Global transcriptome assessment of porcine MII oocytes and IVV embryos. **A** Schematic showing the preparation of porcine MII oocytes and IVV embryos for RNA-seq. For MII oocytes were collected by in vitro maturation; 2-cell, 4-cell, 8-cell and blastocyst embryos were collected by in vivo fertilization (IVV). RNA sequencing was performed using pools of 10 oocytes/embryos (3 replicates per group). **B** Principal component analysis was performed for all developmental stages with their replicates (MII, *n* = 3; 2-cell to blastocyst, *n* = 3). Each point represents a replicate. Developmental stages were clearly separated from each other and replicates within stages were grouped together. **C** Heatmaps showing the gene expression levels among oocytes, 2-cell, 4-cell, 8-cell and blastocyst embryos. Each column represents a replicate. **D** The scatter plots show the upregulation and downregulation DEGs between porcine IVV and SCNT embryo at the 2-cell, 4-cell and 8-cell stage embryos (FC > 2, FDR < 0.05). **E** Cluster analysis showing the gene expression levels form porcine MII oocytes to blastocysts

MII oocytes and in vivo fertilized embryos (2-cells, 4-cells, 8-cells and blastocysts) were collected for subsequent RNA-sequencing analysis and differential analysis of gene expression was performed (Fig. 1A). Principal component analysis showed that the biological replication in each experimental group was good and MII oocytes were most similar to 2-cell embryos, which proved the reliability of the data (Fig. 1B). The profiles of gene expression at various stages (Fold change, FC > 2; False discovery rate, FDR < 0.05) showed dynamic changes (Fig. 1C).

Compared with other stages, the gene expression patterns of MII oocytes and 2-cell embryos were similar and 380 upregulated DEGs and 1639 downregulated DEGs (Fig. 1D) were identified, indicating that the IVV embryos were still under maternal regulation at this time. The gene expression pattern was significantly increased at the 4-cell stage and 3093 upregulated DEGs and 1676 downregulated DEGs were identified when compared with the 2-cell stage, and then decreased from the 8-cell to blastocyst stage, which indicated that the early embryo ZGA of pigs occurred at the 4-cell stage (Fig. 1D). The embryos began the transition from maternal to zygotic regulation at this time. The parallel coordinated expression analysis showed the changes in gene expression patterns from 2-cell and 8-cell embryos (Fig. 1E).

### Functional enrichment analysis of differentially expressed genes in porcine IVV embryos

**Fig. 2:**
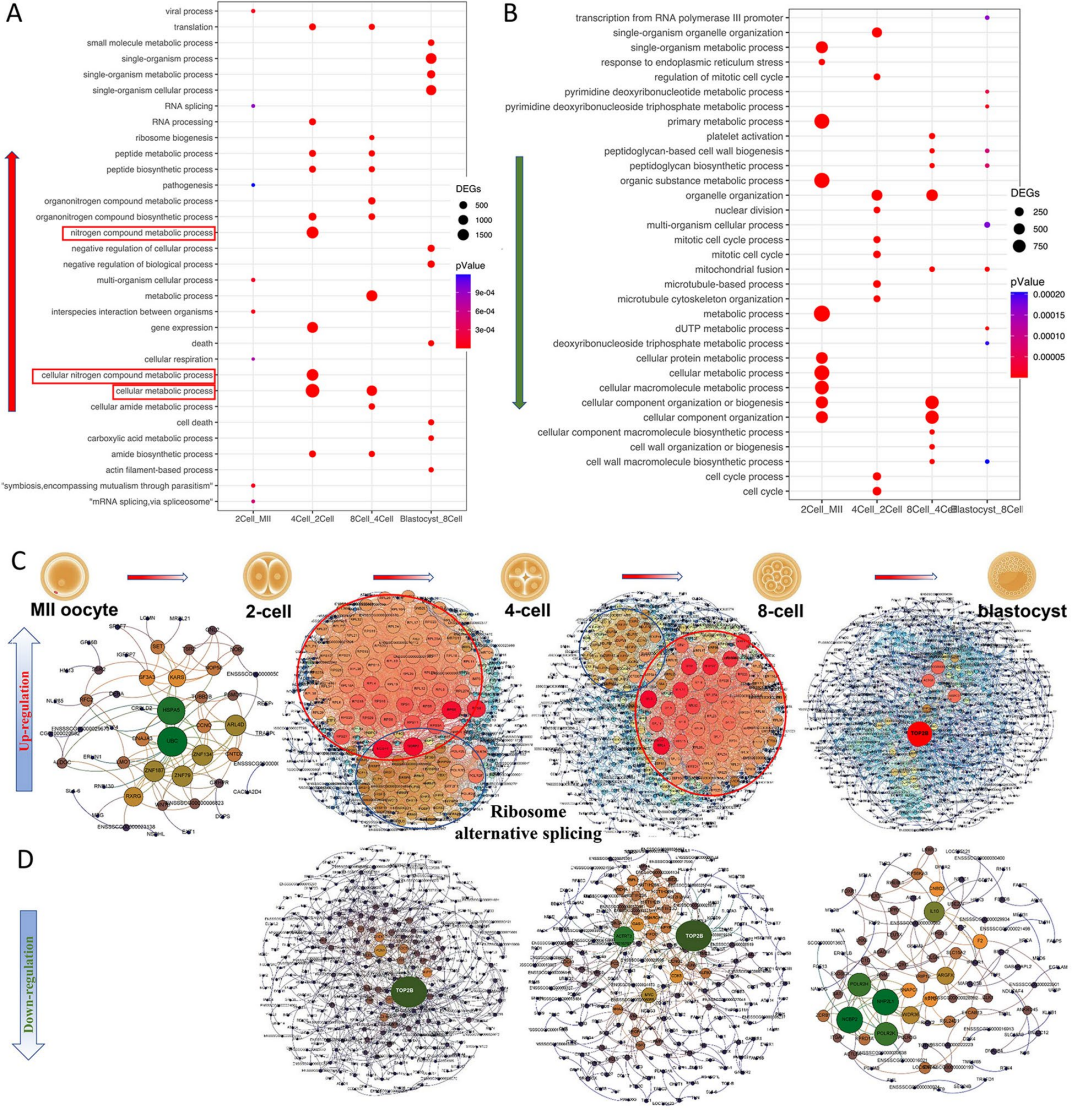
Enrichment analysis and PPI network of porcine DEGs between MII oocytes and IVV embryos. **A**, **B** The enrichment analysis of DEGs and clustering of the upregulated and downregulated DEGs in biological processes. **A** Red arrows represent canonical pathways of DEGs with upregulated and (**B**) green arrows represent canonical pathways of DEGs with downregulated among MII oocytes, 2-cell, 4-cell, 8-cell and blastocyst stages. **C**, **D** PPI network of upregulated and downregulated DEGs among MII oocytes and embryos, at the 2-cell, 4-cell, 8-cell and blastocyst stage

GO enrichment analysis of DEGs at various stages was performed. In terms of biological processes, the upregulated DEGs from the 2-cell to 4-cell stage were enriched in 10 biological processes, which were mainly related to nitrogen compound metabolic process. The upregulated DEGs from the 4-cell to 8-cell stage were enriched in 10 biological processes, which also were mainly related to metabolic process. The upregulated DEGs from the 8-cell to blastocyst stage were enriched in 10 biological processes, which were mainly related to single organism process (Fig. 2A). Meanwhile, the downregulated DEGs from MII oocytes to 2-cell stage were enriched in 10 biological processes, which were mainly related to metabolic process. The downregulated DEGs from the 2-cell to 4-cell stage were mainly enriched in organelle organization. The downregulated DEGs from the 4-cell to 8-cell stage were mainly enriched in cellular compound organization. The downregulated DEGs from the 8-cell to blastocyst stage were enriched in biosynthetic process and mitochondrial fusion (Fig. 2B).

To further clarify the function of DEGs, the Protein-Protein Interaction network (PPI network) analysis of the DEGs was performed in the STRING database in early porcine embryos. The core gene clusters in the PPI network showed that the most closely interacting genes among the upregulated genes during the 2-cell to 4-cell stage were related to ribosome alternative splicing, and the core gene were *NCBP1* and *NCBP2* (Fig. 2C). The core gene among the downregulated genes during the 2-cell to 4-cell stage was *TOP2B* (Fig. 2D).

### Comparative analysis of transcriptome activity between porcine SCNT and IVV embryos

**Fig. 3:**
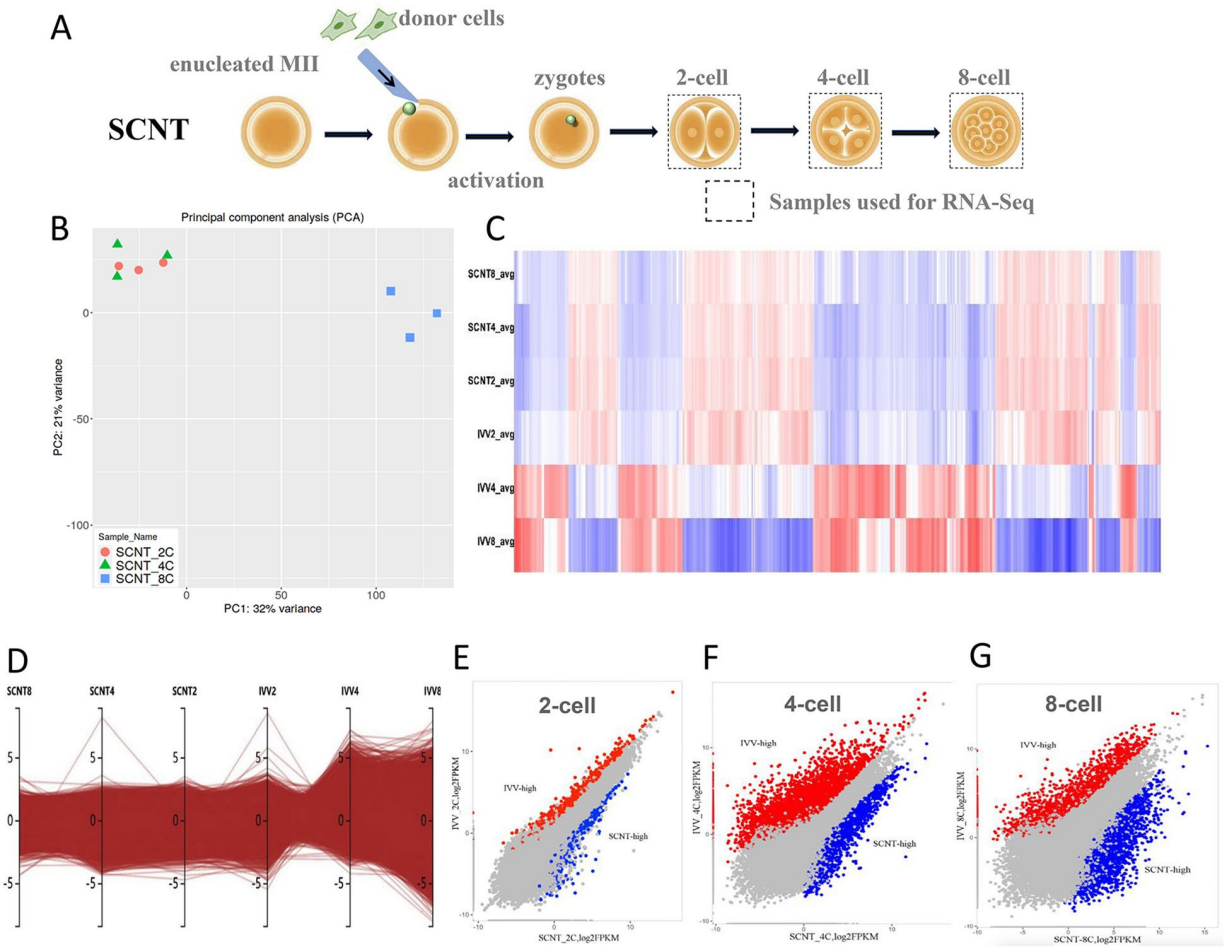
Comparative analysis of transcriptome activity between porcine SCNT and IVV embryos. **A** Schematic showing the preparation of porcine SCNT embryos for RNA-seq. For 2-cell, 4-cell and 8-cell embryos were collected by SCNT. RNA sequencing was performed using pools of 10 oocytes/embryos (3 replicates per group). **B** Principal component analysis was performed for all developmental stages with their replicates. Each point represents a replicate. **C**, **D** Heatmaps and cluster analysis showing the global gene expression levels between SCNT and IVV embryos at the 2-cell, 4-cell and 8-cell stages. **E** Volcano plot of differentially expressed genes (DEGs) between porcine IVV 2-cell and MII oocytes stage, 4-cell and 2-cell stage embryos, 8-cell and 4-cell stage embryos, and blastocyst and 8-cell stage embryos (FC > 2, FDR < 0.05)

To compare the transcriptomic profiles of IVV embryos and SCNT embryos during ZAG, SCNT embryos (2-cells, 4-cells and 8-cells) were collected for RNA-sequencing analysis (Fig. 3A). Principal component analysis showed that each experimental group was well biologically replicated, which proved the reliability of the data (Fig. 3B). The transcriptomic data of SCNT embryos showed that there was little difference in gene expression patterns at the 2-cell and 4-cell stage, which was significant different from the transcriptome dynamic changes in IVV embryos, so there was a ZGA barrier at the 4-cell stage of SCNT embryos (Fig. 3C, D).

Transcriptome data at the 2-cell stage showed that 1484 genes had more than two-fold differences in expression between the IVV and SCNT embryos (FC > 2, FDR < 0.05) (Fig. 3E). There were 5821 genes with differential expression levels of more than 2 times (FC > 2, FDR < 0.05) at the 4-cell stage and 2234 upregulated DEGs and 3587 downregulated DEGs were identified in SCNT embryos, compared with IVV embryos (Fig. 3F). In addition, 5605 genes with more than 2-fold expression levels were screened between the two embryos at the 8-cell stage (Fig. 3G). The results indicated that abnormal zygotic gene activation occurred in the 4-cell stage of porcine SCNT embryos, so we focused on the analysis of the DEGs at the 4-cell stage.

### Biological functions analysis of differentially expressed genes at the 4-cell stage

**Fig. 4:**
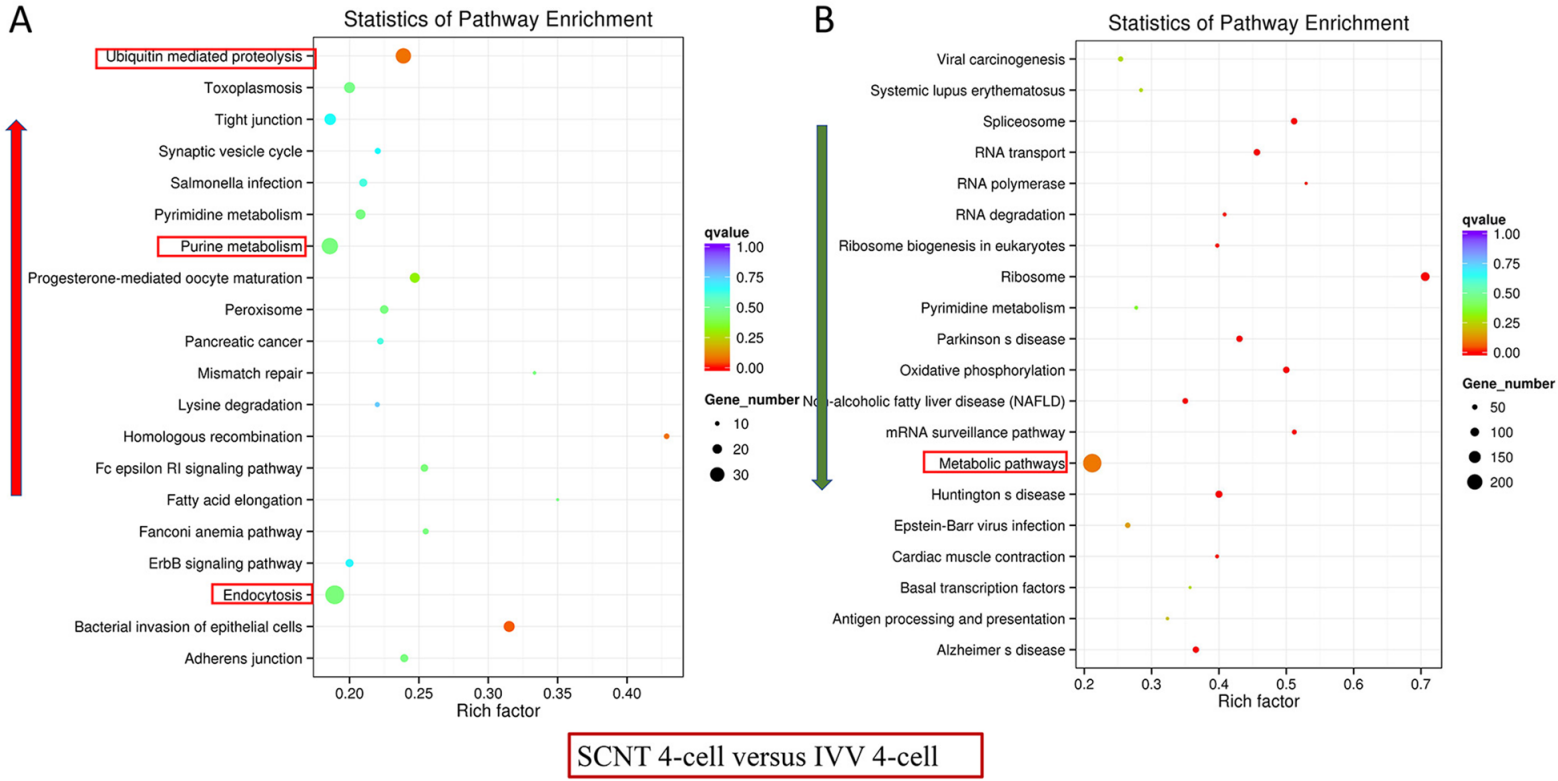
KEGG enrichment analysis of porcine DEGs between SCNT and IVV embryos at the 4-cell stage. **A** Red arrows represent canonical pathways of DEGs that were significantly higher expressed and **B** green arrows represent canonical pathways of DEGs that were significantly lower expressed between SCNT and IVV embryos at the 4-cell stages

To gain insight into the biological functions of the DEGs, a canonical KEGG pathway enrichment analysis was conducted. The persistent difference between IVV and SCNT embryos at the 4-cell stage were illustrated by an enrichment of four canonical pathways (Fig. 4). Except for a higher expression in SCNT versus IVV embryo of DEGs involved in ubiquitin mediated proteolysis, purine metabolism and endocytosis (Fig. 4A), the DEGs involved in metabolic pathways were lower expressed in SCNT than in IVV embryos (Fig. 4B).

### Identification of critical transcription factors in porcine embryos during ZGA

**Fig. 5:**
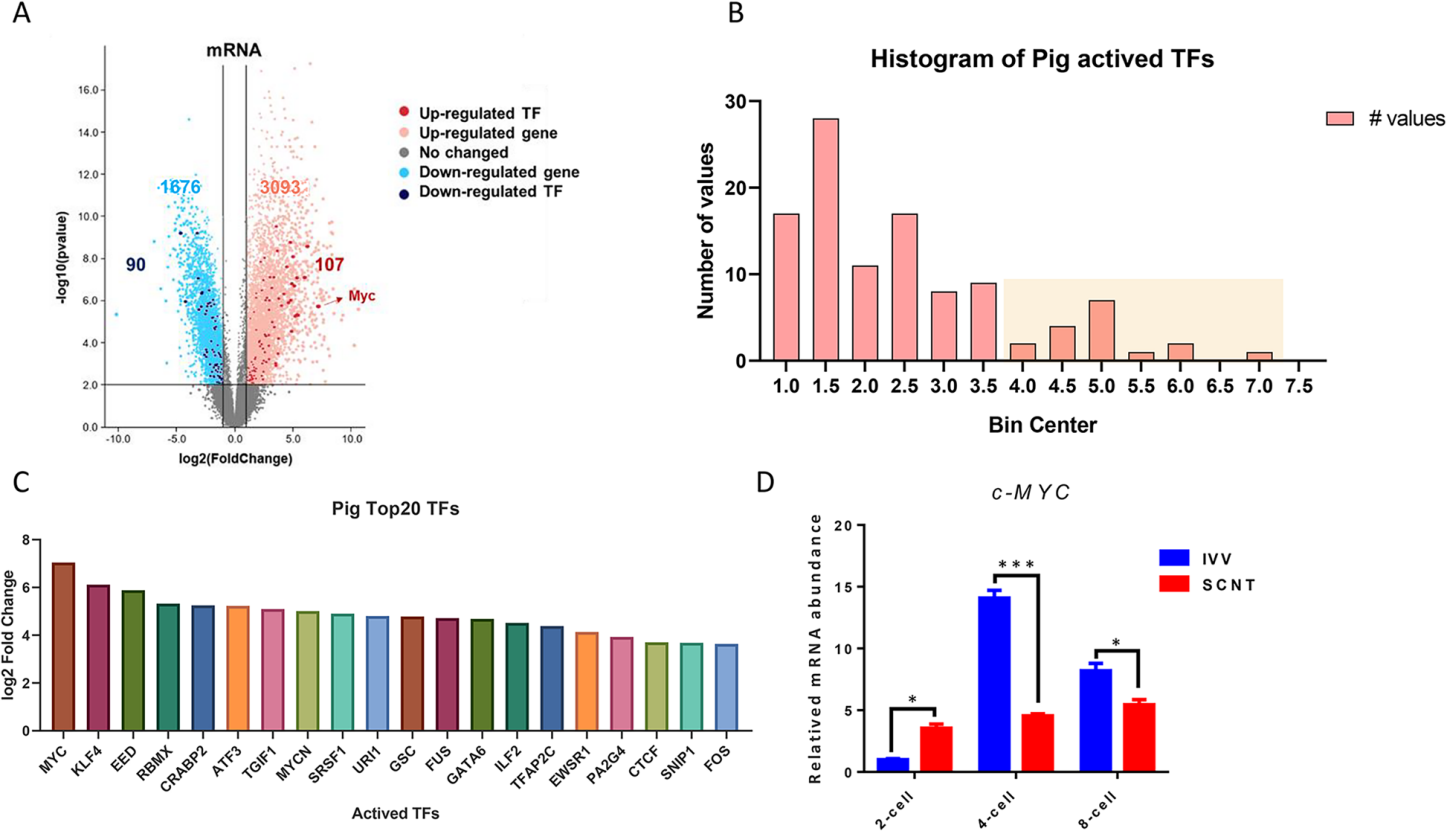
Expression patterns of transcription factors in porcine embryos during ZGA. **A** Volcano plot of upregulated and downregulated DEGs and transcription factors (TFs) of porcine embryos during the ZGA. **B** Probability distribution of expression fold changes of all activated TFs in porcine embryos. **C** List of the top 20 activated TFs in porcine embryos. **D** Relative abundance of *c-MYC* in the porcine IVV and SCNT embryos, quantities were normalized to GAPDH abundance. Data are presented as the mean ± standard deviation. *, *P* < 0.05; **, *P* < 0.01 between groups

We analyzed the TFs of the DEGs during the ZGA stage, and found 107 upregulated and 90 downregulated TFs (Fig. 5A). The probability distribution of fold change in the expression of all activated transcription factors was conducted (Fig. 5B), and the top 20 TFs were listed according to their expression multiple, namely *c-MYC*, *KLF4*, *EED*, *RBMX*, *CRABP2*, *ATF3*, *TGIF1*, *MYCN*, *SRSF*, *URL1*, *GSC*, *FUS*, *GATA6*, *ILF2*, *TFAP2C*, *EWSR1*, *PA2G4*, *CTCF*, *SNIP1* and *FOS*. We found that the expression levels of *c-MYC*, *KLF4*, *EED*, *RBMX*, *CRABP2*, *ATF3* and *TGIF1* were 5-fold higher and that *c-MYC* showed the highest fold expression change in upregulated TFs (Fig. 5C), which might play a critical role in porcine ZGA. The expression levels of *c-MYC* in IVV and SCNT embryos were verified by qPCR, and *c-MYC* had the significantly lower expression level in SCNT embryos, especially in the 4-cell stage (Fig. 5D). It is speculated that *c-MYC* may be an important transcription factor affecting zygotic gene activation.

### Treatment with 10,058-F4 impeded the developmental competence of porcine embryos

**Fig. 6:**
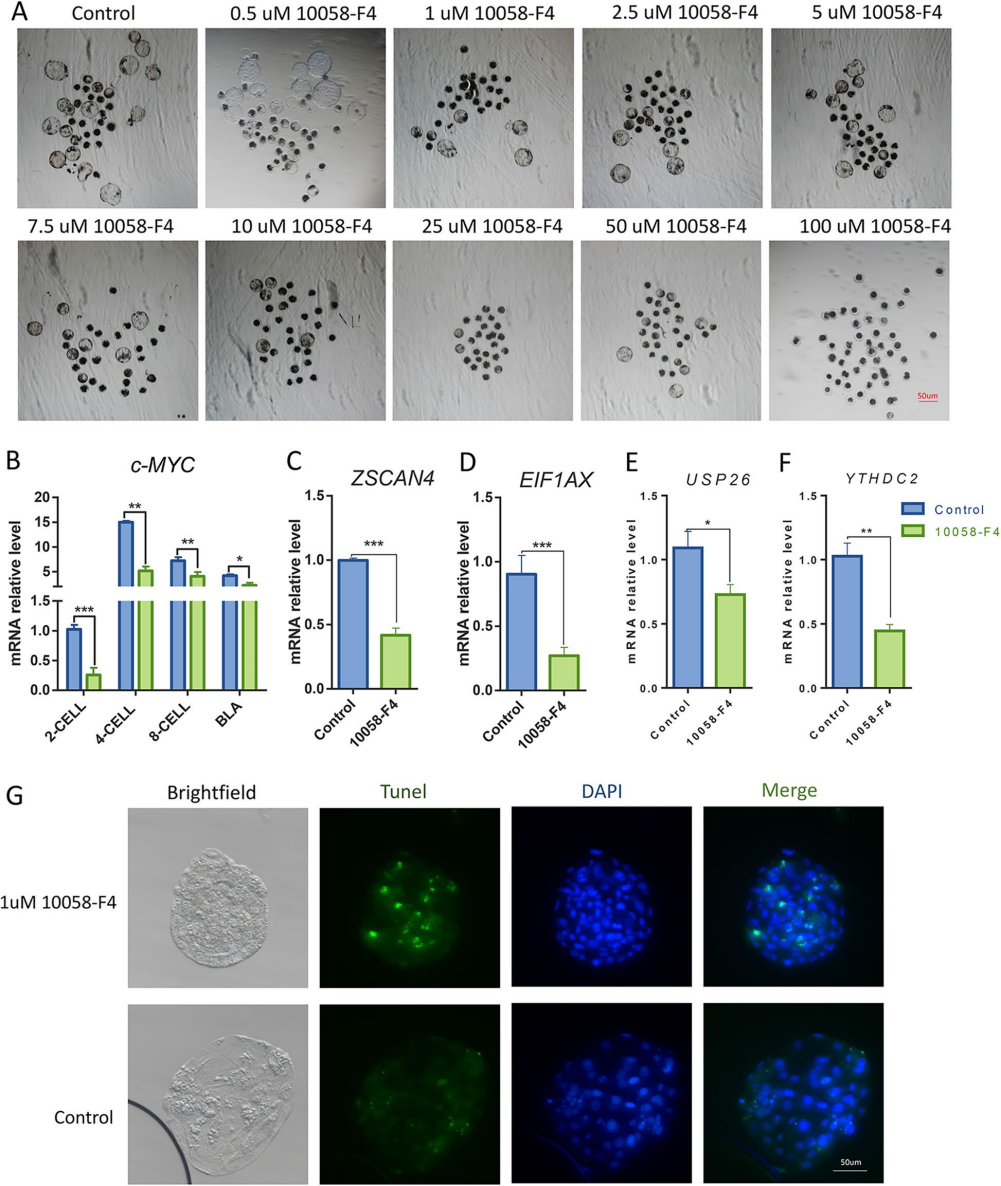
Assessment of the development competence of porcine embryo after treatment with 10,058-F4. **A** Representative images of IVF embryos after culturing for 7 days by treatment with 10,058-F4 at 0, 0.5 uM,1 uM, 2.5 uM, 5 uM, 7.5 uM, 10 uM, 25 uM, 50 uM or 100 uM for 48 h, Scale bars, 50 mm. **B** Relative abundance of *c-MYC* (**B**) and ZGA-related genes *ZSCAN4* (**C**), *EIF1AX* (**D**), *USP26* (**E**) and *YTHDC2* (**F**) in the porcine IVF embryos, quantities were normalized to GAPDH abundance. Data are presented as the mean ± standard deviation. *, *P* < 0.05; **, *P* < 0.01 between groups, as indicated. BLA, blastocyst. **G** Representative examples of fragmented nuclei (green), which are indicative of apoptosis, versus total nuclei (blue), Scale bar, 50 um

To verify the role of *c-MYC* in porcine embryonic development, porcine IVF embryos were treated with 10,058-F4 at 0, 0.5 uM, 1 uM, 2.5 uM, 5 uM, 7.5 uM, 10 uM, 25 uM, 50 uM or 100 uM for 48 h after fertilization. The cleavage rate and blastocyst rage of porcine IVF embryos were all significantly decreased (Table 1, Fig. 6A). Next, we studied the effects of the duration (0, 24 h, 48 h or 72 h) and 10,058-F4 treatment concentration (1 uM) on embryonic development capacity. Treatment with 10,058-F4 for 48 h or 72 h remarkably decreased the cleavage rate and blastocyst rate of IVF embryos (Table 2). Therefore, we applied 1 uM 10,058-F4 for 48 h treatment in subsequent experiments.

**Table 1:** Effect of different concentrations of 10,058-F4 on the developmental competence of porcine embryo

10,058-F4 Treatment (uM)	No. of embryos	No. of embryos cleaved (% ± SEM)	No. of blastocysts (% ± SEM)
0	150	76(50.8 ± 1.34)^A^	51(34.0 ± 0.21)^A^
0.5 uM	145	72(49.7 ± 1.58)^B^	47(32.4 ± 1.94)^A^
1 uM	150	57(38.0 ± 2.34)^B^	35(23.3 ± 1.31)^B^
2.5 uM	150	50(33.3 ± 0.17)^C^	38(25.3 ± 2.47)^B^
5 uM	155	63(40.6 ± 2.09)^B^	46(29.7 ± 2.11)^B^
7.5 uM	155	48(30.9 ± 1.12)^C^	38(24.5 ± 0.45)^B^
10 uM	150	48(32.0 ± 0.91)^C^	31(20.7 ± 1.71)^B^
25 uM	150	47(31.3 ± 2.21)^C^	25(16.7 ± 2.19)^C^
50 uM	150	32(21.3 ± 1.41)^D^	8(5.3 ± 0.98)^D^
100 uM	140	0(0.0 ± 0.00)^E^	0(0.0 ± 0.00)^E^

Values in the same column with different superscripts from A to E differ significantly (*P* < 0.05)

**Table 2:** Effect of time duration of 10,058-F4(1μM) treatment on the developmental capacity of porcine embryo

Time duration (h)	No. of embryos	No. of embryos cleaved (% ± SEM)	No. of blastocysts (% ± SEM)
0	150	77(51.3 ± 2.23)^A^	53(35.3 ± 0.67)^A^
24 h	150	68(45.3 ± 1.11)^A^	47(31.3 ± 1.47)^A^
48 h	150	56(37.3 ± 0.43)^B^	34(22.7 ± 2.13)^B^
72 h	150	58(38.7 ± 0.67)^B^	37(24.7 ± 1.63)^B^

Values in the same column with different superscripts (A and B) differ significantly (*P* < 0.05)

### Treatment with 10,058-F4 interfered with the transcript of ZGA-related genes and damaged the quality of porcine embryos

Compared with SCNT embryos, the development process of IVF embryos is closer to that of in vivo embryos [26, 34]. Therefore, we selected IVF embryos as a model to verify the effect of 10,058-F4 on early embryo development. We used qPCR to detect the expression changes of *c-MYC*. Our results showed that *c-MYC* had a significantly lower expression in the IVF embryos treated with 10,058-F4 compared with control embryos from the 2-cell to blastocyst stage (Fig. 6B). Differences in the expression of ZGA-related genes in 4-cell stage were evaluated using qPCR. Our results showed that the expression of *ZSCAN4*, *EIF1AX*, *USP26* and *YTHDC2* (Fig. 6C-F) was remarkably decreased in IVF embryos treated with 10,058-F4 compared with the control IVF embryos at the 4-cell stage, indicating that 10,058-F4 treatment impeded embryonic reprogramming during ZGA. To assess the quality of blastocysts, we used the terminal deoxynucleotidyl transferase dUTP nick-end labeling (TUNEL) staining to examine the apoptotic cells of blastocysts. TUNEL staining showed that the apoptotic rate of IVF blastocysts treated with 10,058-F4 was significantly higher than that of the control IVF blastocysts (Fig. 6G), in which 10,058-F4 treatment damaged the quality of blastocysts.

### Transcriptional activities of preimplantation embryos in mice, human and bovine

**Fig. 7:**
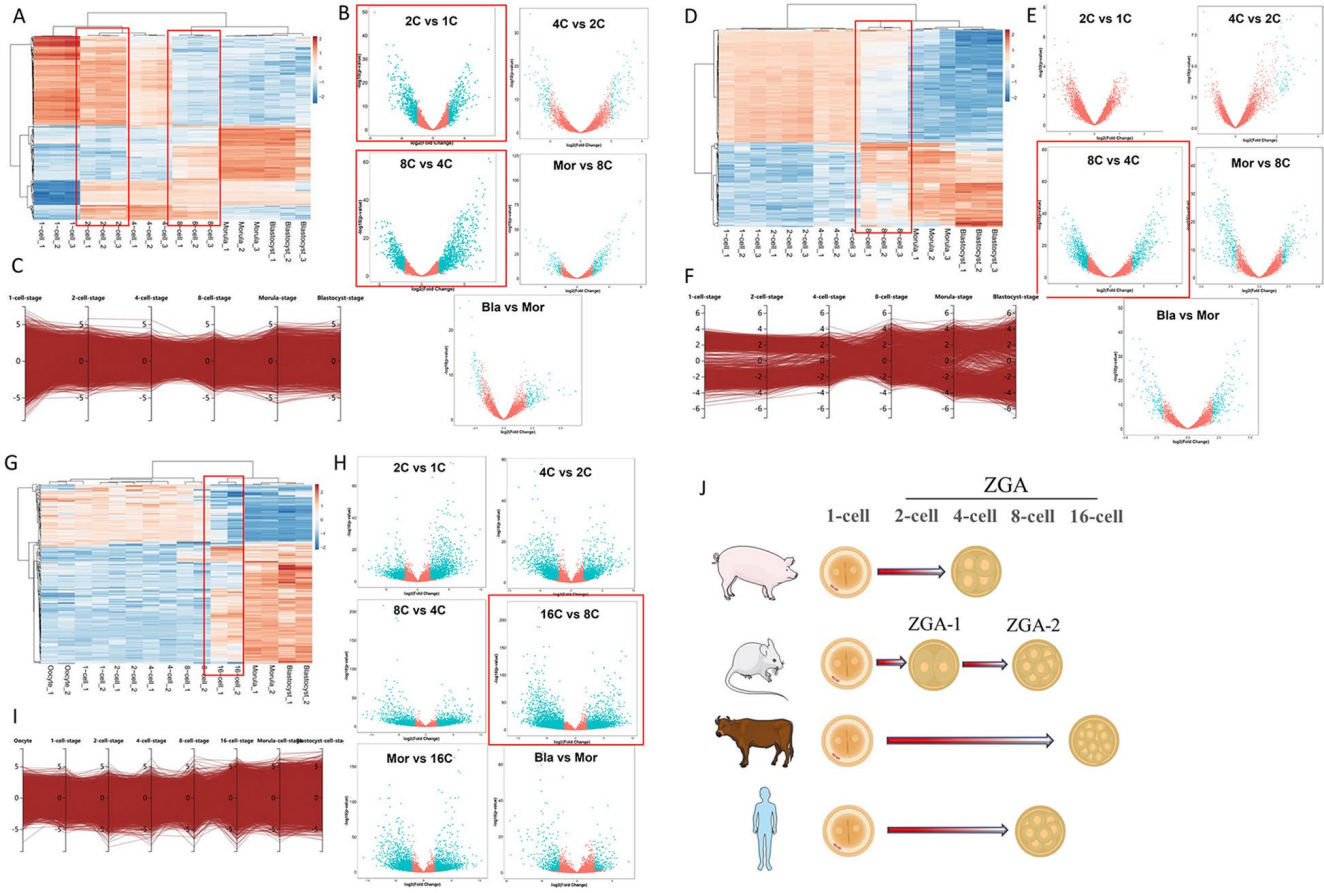
Global transcriptome assessment of preimplantation embryos in mice, human and bovine. **A** Heatmaps showing the gene expression levels of mouse embryos among 1-cell, 2-cell, 4-cell, 8-cell, morula and blastocyst stages. Each column represents a replicate. **B** Volcano plot of differentially expressed genes (DEGs) of mouse embryos between 2C vs. 1C, 4C vs. 2C, 8C vs. 4C, Mor vs. 8C and Bla vs. Mor (FC > 2, FDR < 0.05). **C **The expression of DEGs in mouse embryos during 1-cell to blastocyst stage. **D** Heatmaps showing the gene expression levels of human embryos among 1-cell, 2-cell, 4-cell, 8-cell, morula and blastocyst stage. Each column represents a replicate. **E** Volcano plot of differentially expressed genes (DEGs) of human embryos between 2C vs. 1C, 4C vs. 2C, 8C vs. 4C, Mor vs. 8C and Bla vs. Mor (FC > 2, FDR < 0.05). **F** The expression of DEGs of human embryos from 1-cell to blastocyst stage. **G** Heatmaps showing the gene expression levels of bovine embryo among oocytes, 1-cell, 2-cell, 4-cell, 8-cell, morula and blastocyst. Each column represents a replicate. **H** Volcano plot of differentially expressed genes (DEGs) of bovine embryos between 2C vs. 1C, 4C vs. 2C, 8C vs. 4C, 16C vs. 8C, Mor vs. 16C and Bla vs. Mor (FC > 2, FDR < 0.05). **I** The expression of DEGs in human embryos from oocytes to blastocyst stage. Mor, Morula; Bla, Blastocyst. **J** Schematic overview of the timing of zygotic genome activation in pigs, mice, humans and bovines

We further performed cross-species transcription analysis to analyze the gene expression patterns in mouse, human, and bovine preimplantation embryos. In mice, the numbers of differentially expressed genes began to increase at the 2-cell stage, in which 1802 upregulated DEGs and 1641 downregulated DEGs were identified. The subsequent transformation of gene expression patterns occurred at the 8-cell stage, and 1516 upregulated DEGs and 1287 downregulated DEGs were identified (Fig. 7A, B). The parallel coordinates expression analysis showed the changes of gene expression from 1-cell to blastocyst using edge R (Fig. 7C). Thus, the activation of zygote genes in mice is completed in two stages, 2-cell and 8-cell stage. We named the two ZGAs as mouse-1 and mouse-2 activations in mice.

For humans, the heatmap and parallel coordinated expression analysis showed that the gene expression range change appeared in 8-cell embryos (Fig. 7D-F), and 2158 upregulated DEGs and 2374 downregulated DEGs were identified, indicating that the zygote gene activation of human early embryos occurred at the 8-cell stage.

In bovines, the heatmap and parallel coordinated expression analysis showed that the gene expression range changes appeared in 16-cell embryos (Fig. 7G-I), and 1156 upregulated DEGs and 1025 downregulated DEGs were identified, in which zygote gene activation of bovine early embryos occurred at the 16-cell stage.

Taken together, comparative transcriptome analysis revealed different patterns and timing of zygotic genome activation in different species, with mice even requiring two rounds of zygotic genome activation for the complete reprogramming process (Fig. 7J).

### Functional enrichment analysis of DEGs during ZGA in four species

**Fig. 8:**
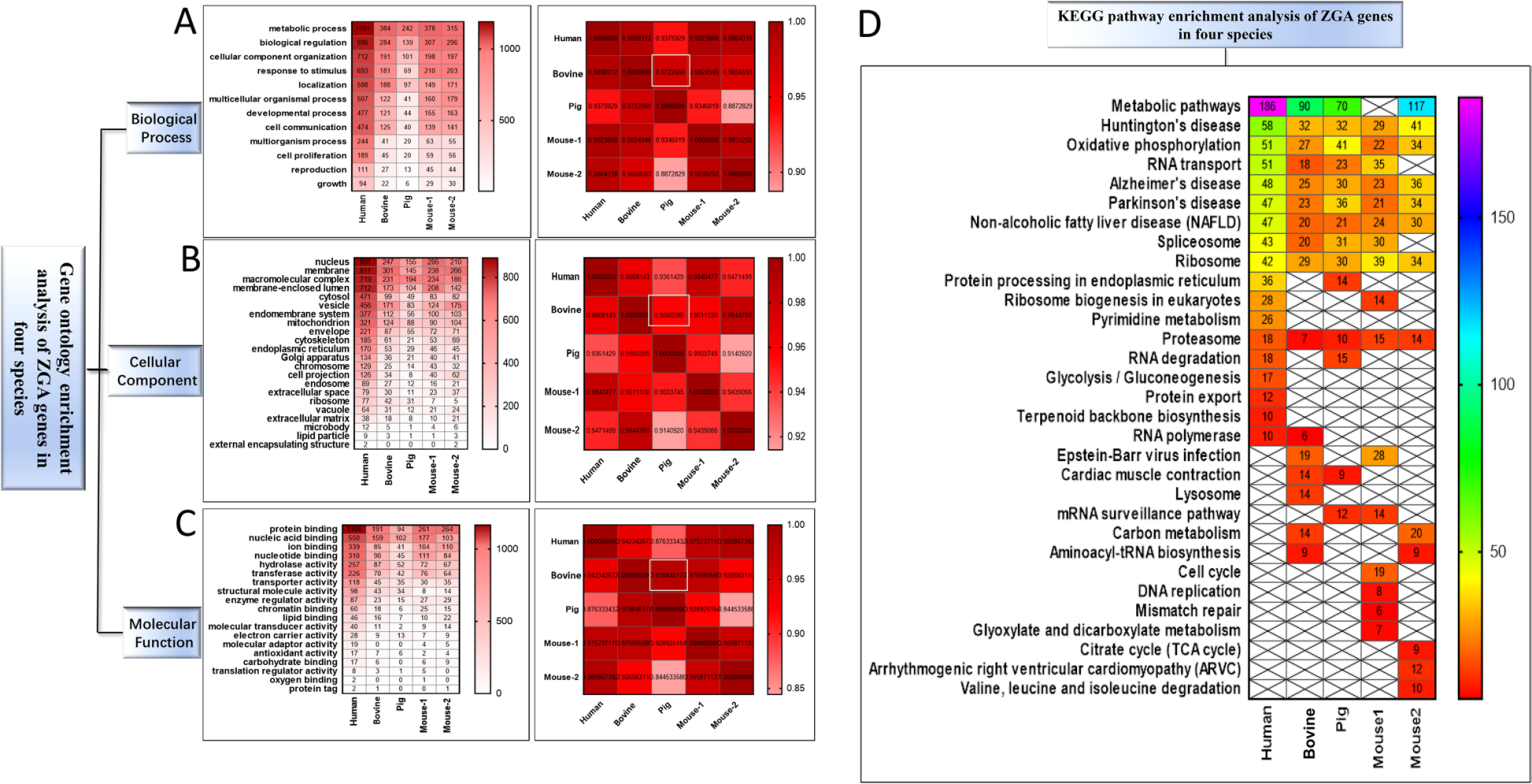
GO and KEGG enrichment analysis of DEGs in pigs, mice, humans and bovines during ZGA. **A** Enrichment of DEGs in biological processes and correlation coefficient of enrichment for four mammals. **B** Enrichment of DEGs in cellular component and correlation coefficient of enrichment for four mammals. **C** Enrichment of DEGs in molecular function and correlation coefficient of enrichment for four mammals. **D** Enrichment of DEGs in the KEGG pathways of four mammals

The cross-species gene ontology (GO) enrichment analysis was performed on the DEGs of the ZGA in four species. In terms of biological process, the DEGs of the ZGA stage in pigs were enriched in 12 biological processes, which were mainly related to metabolic process, biological regulation, cellular component organization, response to stimulus, localization and multicellular organismal process. Among the other three species, bovines had the highest correlation with pigs, with a correlation coefficient of 0.972, followed by humans (0.937) and mice (0.934) (Fig. 8A). In terms of cellular component, the DEGs at the ZGA stage of pigs were enriched in 21 cellular components, mainly related to nucleus, membrane, macromolecular complexes, membrane-enclosed lumen, cytosol, vesicle and mitochondrion. Among the other three species, bovines and pigs were the most closely related, with a correlation coefficient of 0.956, followed by mice (0.950) and humans (0.936) (Fig. 8B). In terms of molecular function, the DEGs at the ZGA stage of porcine embryos were enriched in 15 molecular functions, which were mainly related to protein binding, nucleic acid binding, ion binding, hydrolase activity and nucleotide binding. Among the other three species, bovines had the highest correlation with pigs, with a correlation coefficient of 0.979, followed by mice (0.092) and humans (0.088) (Fig. 8C).

The cross-species KEGG pathway enrichment analysis was performed on the differentially expressed genes of the ZGA in four species. The DEGs were enriched in 14 pathways, and ten of these pathways were shared among the four species. They were metabolic pathways, Huntington’s disease, oxidative phosphorylation, RNA transport, Alzheimer’s disease, Parkinson’s disease, non-alcoholic fatty liver disease, spliceosome, ribosome and proteasome. In addition, each of the four species had its own specific enrichment pathways (Fig. 8D).

### Comparative analysis between total transcripts and differentially expressed genes during zygotic gene activation in four species

**Fig. 9:**
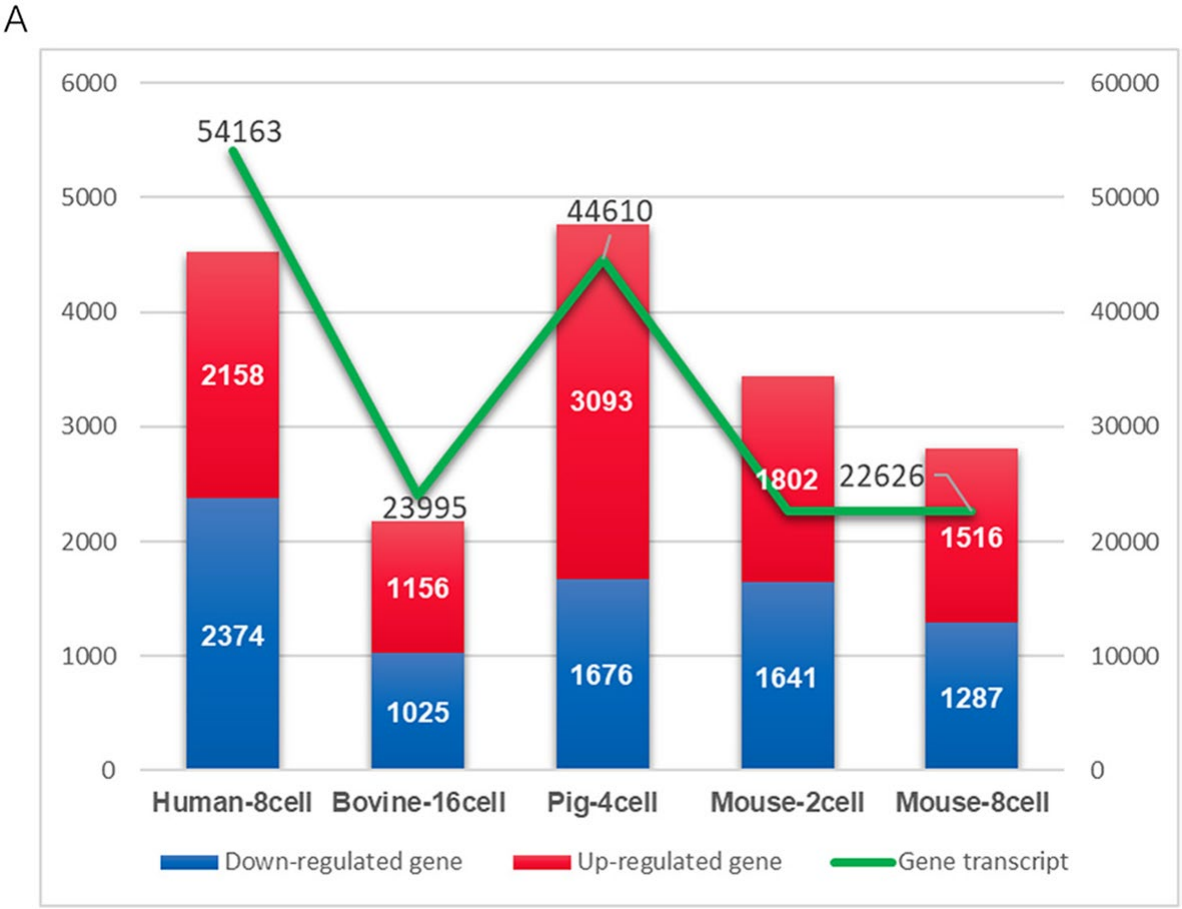
Statistical analysis between total transcripts and differentially expressed genes during ZGA in four species. **A** The numbers of DEGs and total transcripts during the ZGA in human, bovine, pig and mouse. DEGs: Differentially Expressed Genes; ZGA: Zygotic Gene Activation

We analyzed the number of DEGs and total transcripts during ZGA in four species. The total number of DEGs in pigs was 4769, accounting for 10.7% of the total transcripts. The total number of DEGs during the first ZGA in mouse embryos was 3442, accounting for 15.2% of the total transcripts, and a total of 2803 DEGs during the second zygotic activation stage, accounting for 12.4% of the total transcripts. There were 4532 DEGs during the ZGA of human early embryos, accounting for 8.4% of the total scripts. A total of 2181 DEGs in bovines accounted for 9.1% of the total transcripts (Fig. 9A).

## Discussion

In the early stage of mammalian embryo development, the dominant role in regulating development gradually shifts from maternal origin to zygote. The depletion of maternal factors and the activation of zygotic genes combine to reprogram terminally differentiated germ cells into totipotent embryos [35]. Due to the rapid development of high-throughput sequencing technology, it is possible to use this technology to carry out transcriptome studies of early embryos, particularly in large mammals such as pigs and bovines, in which embryos are difficult to collect, to reveal the mechanism of mammalian gene transcription more widely and deeply. In the present study, we revealed the transcriptome profiles of porcine IVV and SCNT embryos and performed cross-species analysis of transcriptional activity during ZGA in pigs, mice, bovines and humans. We identified the top 20 transcription factors that were likely key players in activating the porcine zygotic genome and reported conserved features of the transcriptome of the four species during ZGA, which will broaden the current understanding of transcriptional activation during embryo reprogramming and provide references for future studies.

ZGA, also known as embryonic genome activation, is important for preimplantation embryo development in mammals [36]. It has been reported that when thousands of genes are activated, genome-wide transcription and major ZGA occur [37–39]. Our results investigated the persisting transcriptional differences between in IVV and SCNT during ZGA and understand their relationship to porcine embryo competence. In our study, the 4-cell SCNT embryos displayed a higher transcript expression of genes related to ubiquitin mediated proteolysis, purine metabolism and endocytosis pathways compared with IVV embryos. Ubiquitin mediated proteolysis is an important pathway for efficient and highly selective specific protein degradation, and is widely involved in various metabolic activities. Abnormal protein degradation rates and sites can affect a variety of cellular functions, such as cell division and signaling [40, 41]. Meanwhile, ubiquitination proteolysis performed by RNF8 is critical to the enrichment of DNA damage repair proteins in the DSB region, and thus protects genome integrity and stability [42]. Purine metabolism and endocytosis pathways regulate many basic biological processes of all organisms and play a dominant role in the growth, development, reproduction and inheritance of organisms [43, 44]. The anomalous enrichment of these pathways in SCNT embryos during ZGA have adverse effect on cell reprogramming and subsequent embryo competence.

In addition, 21.4% of genes related to metabolic pathways were displayed to be downregulated in 4-cell SCNT embryos compared to in vivo developed porcine 4-cell embryos. The metabolic pathways main include Tricarboxylic acid cycle (TCA cycle), amino acid metabolism, bile acid metabolism, fatty acid metabolism, acylcarnitine, pentose phosphate metabolism, which participates in normal life activities [45, 46]. As an important metabolic pathway, the TCA cycle provides necessary reaction substrates and energy for genome activation, chromatin opening and epigenetic modification in embryonic ZGA [47]. It is reported that the increased expression levels of fatty acid metabolism related genes could promote the degradation of fatty acids in embryos, provide energy for embryonic development, and improve the ability of porcine embryonic development in vitro [48]. Pentose phosphate pathway is an important branch of aerobic oxidation of sugars and can provide NADPH for biosynthesis, pentose for nucleic acid metabolism, and energy for biological activity through glycolysis [49]. Thus, we propose that the dysregulation of metabolic pathways in SCNT embryo during ZGA results in the inability to provide necessary reaction substrates and energy for genome activation, chromatin opening, and epigenetic reprogramming, which is also detrimental to later embryonic development.

How the zygotic genome is activated during early embryonic development has always been a research hotspot. The activation factors of the genome can bind to silent chromatin regions that are inaccessible to most other DNA-binding factors and mediate increased chromatin accessibility, which plays an important role in activating the zygotic genome to establish embryonic transcription programs [50–52]. It has been reported that a variety of transcription factors have been identified in humans and mice [53, 54]. These transcription factors are essential for activating the zygotic genome and initiating transcription. Researchers found that *Dux* and *Nfya* are the first transcription factors for initiating the embryonic genome and play an important role during ZGA in mice [55, 56]. Transcription factor *OCT4* is necessary for ZGA in human embryos, and the binding sites of *OCT4* are enriched in the open chromatin region of embryos [13]. *OCT4* is activated synchronously with the early activation genes of ZGA, and its protein immediately participates in the subsequent major ZGA [57]. *KLF5*, which potentially regulates the expression of 58% of the embryonically expressed transcripts, plays important roles in reprogramming gene expression in preimplantation bovine embryo development [58]. Liu et al. found that thymine DNA glycosylase was defined as a pig-specific epigenetic regulator for nuclear reprogramming. It has been reported that 10,058-F4, a small molecular inhibitor of *c-MYC*, can inhibit the c-*MYC*-Max interaction and prevent transactivation of c-*MYC* target gene expression [59]. To date, the key transcription factors involved in the activation of the zygotic genome in pigs have rarely been reported. The activated transcription factors were analyzed in our study and *c-MYC* had the highest fold expression change in upregulated TFs, which may play a critical role in porcine ZGA. Our results showed that treatment with 10,058-F4 for 48 h remarkably decreased the developmental competence of porcine embryos and damaged the quality of blastocysts. Meanwhile, the expression levels of the ZGA-related genes *ZSCAN4*, *EIF1AX*, *USP26* and *YTHDC2* were significantly decreased in porcine embryos at the 4-cell stage. Therefor, 10,058-F4 treatment impeded the reprogramming of embryos during ZGA and *c-MYC* helped in the activation of the zygotic genome in pigs. The deeper regulatory mechanism of *c-MYC* on preimplantation development in IVF and SCNT embryos and the role of other transcription factors in ZGA also need to be further studied.

The transcriptome characteristics of the zygotic genome in mice and humans have been reported [25, 30]. Vera et al. found that in vivo developed and in vitro produced porcine embryos displayed largely similar transcriptome profiles in 4-cell embryos, morulae and hatched blastocysts [60]. However, the researcher only observed embryos at three stages and no continuous evidence provided to show the global transcriptome level of porcine embryos. In this study, MII oocytes and in vivo fertilization embryos from the 2-cell, 4-cell, 8-cell and blastocyst stages were collected and transcriptome sequencing was performed strictly according to 3 biological replicates. Principal components analysis showed that the data at each stage of our experimental group had good repeatability, which ensured the reliability of the results. At present, there are few reports on the comprehensive comparison analysis of ZGA in goats, mice, bovines and humans based on high-throughput analysis [4, 61]. This study was the first to comprehensively analyze the transcriptional map of early embryonic development in pigs, mice, bovines and humans, and to elucidate the commonality and specificity of mammalian ZGA and its transcriptional patterns in these four species. By comparing the gene expression patterns at different stages, we found that the transition from maternal to zygote was a highly species-conserved biological process, however each species had its own ZGA time point and activated gene numbers.

## Conclusions

Taken together, we show that the transcriptome profiles at the early development IVV and SCNT embryo display largely different. Comparing to the IVV embryo, the lower expression of transcripts in 4-cell SCNT embryos were mainly enrichment to metabolic pathways, which indicated a lower developmental competence. Embryos with impaired developmental competence may be arrested at an early stage of development. c-MYC helps promote ZGA and preimplantation embryonic development of pigs. Comparative analysis of pigs, mice, bovines and humans indicates that zygotic genome activation is a process of accumulating energy for large-scale gene transcription, and pigs and bovines have the highest similarity coefficient.

## Materials and methods

### Chemicals

All chemicals and reagents were purchased from Sigma Aldrich (St. Louis, MO, USA), unless otherwise noted. 10,058-F4 was purchased from Selleck Chemicals (Houston, TX, USA).

### Collection of porcine MII oocytes, IVV embryos and SCNT embryos

MII oocytes were collected after 48 h of in vitro maturation, as previously described [62]. To obtain IVV embryos, female Large White pigs were artificially fertilized twice at intervals of 24 hours. The oviduct and uterus were surgically rinsed with PBS at 24 hours, 48 hours, 72 hours, and 96 hours after the first artificial insemination, and the embryos that developed to the 2-cell, 4-cell, 8-cell and blastocyst stages were collected. SCNT was conducted according to previously described protocols [62]. The first polar body of matured oocytes was removed under an inverted microscope and using the blind-suction method. Approximately 10% of the cytoplasm, including the nucleus, was removed in drops of 5 μg/mL cytochalasin B. Then, porcine FFs was injected into the perivitelline space of the enucleated-oocytes and got the reconstructed cloned embryo. The reconstructed embryos were cultured for 1 h in PZM3 and then activated by two successive direct-current pulses at 1.45 kV/cm for 100 μ sec using a CF-150B electro-fusion instrument (CF-150B, BLS, Hungary). The activated cloned embryos were then continuously cultured in PZM-3 medium, at 38.5 °C, 5% CO2 and 100% humidity. The SCNT embryos developed to the 2-cell, 4-cell and 8-cell stages were collected.

All embryos were treated with acid Tyrode’s solution (pH 2.5) for 30–60 s to remove the zona pellucida at room temperature. Then the embryos were washed repeatedly in PBS containing 1% BSA to remove the Tyrode’s solution and the cell debris. Finally, at least three embryos were collected in 2.5 μl precooled cell lysis buffer in each group with a small volume of PBS. The total volume was kept within 3.5 μl and stored at − 80 °C. The cell lysis buffer containing samples was transported to the sequencing company as quickly as possible by storing in a sufficient amount of dry ice. Each group of samples was guaranteed to have 3 biological repeats.

### RNA sequencing (RNA-seq) of porcine embryos and MII oocytes

The entire sequencing process of this experiment was commissioned to Beijing Novogene Biological Company (Beijing, China), including the extraction and quality control of RNA samples, cDNA amplification and library establishment, and computer sequencing. mRNA was purified from total RNA using poly-T oligo-attached magnetic beads. Fragmentation was carried out using divalent cations under elevated temperature in NEB Next First Strand Synthesis Reaction Buffer (5X). First strand cDNA was synthesized using random hexamer primers and M-MuLV Reverse Transcriptase. RNase H-. Second strand cDNA synthesis was subsequently performed using DNA Polymerase I and RNase H. Remaining overhangs were converted into blunt ends via exonuclease/polymerase activities. After adenylation of the 3′ ends of DNA fragments, NEB Next Adaptors with hairpin loop structures were ligated to prepare for hybridization. To preferentially select cDNA fragments 150 ~ 200 bp in length, the library fragments were purified with an AMPure XP system (Beckman Coulter, Beverly, USA). Then, 3 μl USER Enzyme (NEB, USA) was used with size-selected, adaptor-ligated cDNA at 37 °C for 15 min followed by 5 min at 95 °C before PCR. Then, PCR was performed with Phusion High-Fidelity DNA polymerase, Universal PCR primers and Index (X) Primer. Finally, PCR products were purified (AMPure XP system) and library quality was assessed on the Agilent Bioanalyzer 2100 system. The clustering of the index-coded samples was performed on a cBot Cluster Generation System using TruSeq PE Cluster Kit v3-cBot-HS (Illumina) according to the manufacturer’s instructions. After cluster generation, the library preparations were sequenced on an Illumina HiSeq platform and 125 bp/150 bp paired-end reads were generated.

Raw sequence data are available at Genome Sequence Archive (GSA) database (https://ngdc.cncb.ac.cn/gsub/submit/gsa/list), and the assigned accession of the submission is: CRA006174, detail accession numbers: MII_1 (CRX404164), MII_2 (CRX404165), MII_3 (CRX404166), IVV_2C_1 (CRX335537), IVV_2C_2 (CRX335538), IVV_2C_3 (CRX335539), IVV_4C_1 (CRX335540), IVV_4C _2 (CRX335541), IVV_4C_3 (CRX335542), IVV_8C_1 (CRX335543), IVV _8C_2 (CRX335544), IVV_8C_3 (CRX335545), IVV_Bla_1 (CRX335546), IVV_Bla_2 (CRX335547), IVV_Bla_3 (CRX335548), SCNT_2C1 (CRX335549), SCNT_2C2 (CRX335550), SCNT_2C3 (CRX335551), SCNT_4C1 (CRX335552), SCNT_4C2 (CRX335553), SCNT_4C3 (CRX335554), SCNT_8C1 (CRX335555), SCNT_8C2 (CRX335556), SCNT_8C3 (CRX335557).

### Differential expression profiling

Differential expression analysis was performed using the DESeq R package (1.18.0). DESeq provides statistical routines for determining differential expression in digital gene expression data using a model based on the negative binomial distribution. The resulting *P*-values were adjusted using Benjamini and Hochberg’s approach for controlling the false discovery rate. Genes with an adjusted *P*-value < 0.05 found by DESeq were considered differentially expressed. Degust (http://vicbioinformatics.com/degust/), an interactive web tool for visualizing differential gene expression data, was used to generate the parallel coordinate plot (PCP) based on the data from RNA-Seq [63]. The heatmap showing the genic relationship matrices were created in R studio Version 1.1.383 (https://www.R-project.org/) with the heatmap package (https://CRAN.R-project.org/package=heatmaply). Complete linkage hierarchical clustering was performed by the Euclidean distance measure. RNA-seq datasets of early embryos in mice, humans and bovines, were downloaded from the Gene Expression Omnibus database. The mouse and human RNA-seq dataset (GSE18290) contains sequence read archive (SRA) files of one cell stage, two cell stage, four cell stage, eight cell stage, morula, and blastocyst. Mouse embryos were obtained by flushing from the oviducts and individual one cell stage, two cell stage, four cell stage, eight cell stage, morula, and blastocyst embryos were morphologically staged by light microscopy. Human embryos were obtained from patients at the Boston IVF Clinic through informed consent with the approval of the Internal Review Board of Harvard University and all duly obtained by full review of an ESCRO committee and IRB with certified consent forms. Embryos thus obtained were cultured in a two-step culture system (Sage/Biopharma) in microdrops at 37 °C and 5% CO_2_ under oil (Sage) as described previously [64]. Human embryos from one cell stage, two cell stage, four cell stage, eight cell stage, morula, to blastocyst were collected. Bovine RNA-seq dataset (GSE18290) contains sequence read archive (SRA) files of oocyte, one cell, two cell, four cell, eight cell, sixteen cell, morula, and blastocyst stage. Bovine oocytes were collected by in vitro maturation culture system in 5% carbon dioxide and air (39 °C, high humidity). Bovine one cell, two cell, four cell, eight cell, sixteen cell, morula, and blastocyst embryos were obtained by in vitro fertilization [61].

### In vitro fertilization (IVF) of porcine oocytes

IVF was conducted according to previously described protocols [65]. Frozen porcine semen was purchased from Hebei Mingge Animal Husbandry Co. Ltd. (Hebei, China). The frozen semen was quickly thawed. Groups of 20 oocytes were transferred to 50 μl of mTBM covered with paraffin oil. The final sperm concentration was 2 × 10^6^ sperm/ml. The oocytes were cocultured with sperm for 6 hours at 38.5 °C with 5% CO_2_, and then the oocytes were transferred to porcine zygote medium 3 (PZM3) for continued culture. More than 200 embryos were constructed each time.

### GO and KEGG enrichment analysis of differentially expressed genes

Gene ontology (GO) analysis of the four clusters in pigs, mice, bovines and humans was conducted separately using the R package cluster profiler (v3.18.1), Bioconductor annotation data package org. Hs.eg.db (v3.12.0) and org.Mm.eg.db (v3.12.0). GO terms with a false discovery rate (FDR) adjust *P*-value < 0.05 were deemed statistically significant.

KEGG analysis of the four clusters in pigs, mice, bovines and humans was conducted separately using KOBAS software to test the statistical enrichment of differentially expressed genes in KEGG pathways [66].

### PPI analysis of differentially expressed genes

PPI analysis of differentially expressed genes was based on the STRING database, which known and predicted Protein-Protein Interactions. For the species existing in the database, we constructed the networks by extracting the target gene list from the database; Otherwise, Blastx (v2.2.28) was used to align the target gene sequences to the selected reference protein sequences, and then the networks were built according to the known interactions of selected reference species.

### Immunofluorescence (IF) staining

There were more than 10 embryos in each group for IF staining, which was repeated no fewer than three times. The zona pellucida of blastocysts was digested using 0.5% pronase in PBS. The zona pellucida-free blastocysts were fixed for 30 min at RT with 4% paraformaldehyde, permeabilized for 30 min with 0.2% Triton X-100, and then blocked for 1 h at 37 °C with 1% BSA (w/v) in PBS. The blastocysts were incubated for 1 h at 37.5 °C with primary antibodies (TUNEL, 11684817910) (Roche, Basel, Switzerland). The DNA was stained for 10 min with 10 μg/mL DAPI prior to mounting and observation under a fluorescence microscope (Nikon, Tokyo, Japan).

### RNA isolation and quantitative PCR

RNA isolation and quantitative PCR were conducted according to previously described protocols [62]. The REPLI-g® WTA single cell kit (Qiagen, Hilden, Germany) was used to extract the total RNA and synthesize cDNA from 200 of porcine embryos. The primers used are listed in Table 3. Additionally, we defined gene expression as the cutoff when the Ct mean reached 35. We compared the expression of different reference genes (*GAPDH*, *B2M*, *H2A* and *ACTB*) in different developmental stages of porcine oocytes and early IVF embryos. The results showed that the expression of the GAPDH gene was relatively stable in oocytes and early embryos, and there was no significant difference between them (Supplementary Fig. 1). Therefore, we chose GAPDH as a reference in this research.

**Table 3:** Primers used in the qPCR analysis

Gene	Sequences (5′-3′)	Fragments size	Genebank ID
*ZSCAN4*	F: *TGTTCCCAGGTCTTCCGATAT* R: *TCAGGTGGCGGTTGTAGGT*	298	XM_021097584.1
*c-MYC*	F: CTGAGGCACACAAAGACT R: GCTTGGACAGGTTAGGAG	105	NM_001005154.1
*YTHDC2*	F: *GTACGGTTTCATCCCACT* R: *TAGGAATCCCATCTGCTC*	165	XM_021084627.1
*EIF1AX*	F: *CCAAGAATAAAGGTAAAGGAGGTA* R: *ATCGTCCATTTCCCAGCA*	*109*	NM_214380.2
*USP26*	F: *ACCAGCAGCACATGTCAAGA* R: *CGCCTGTTCAGACTCTTGGT*	201	XM_021080583.1
*GAPDH*	F: CAAATTCATTGTCGTACCAG R: ACACTCACTCTTCTACCTTTG	102	NM_001206359.1

### Statistical analysis

All experiments were replicated at least three times. Data are presented as the mean ± standard error of mean. The statistical analysis between two groups was carried out by two-tailed Student’s t-test using SPSS (Statistics Production for Service Solution) v19.0 software (Chicago, IL, USA) and one-way ANOVA among three or more groups. *P*-value < 0.05 was considered statistically significant, and *P*-value < 0.01 was considered extremely significant.

## Supplementary Information

**Additional file 1: Supplementary Fig. 1.** Expression patterns of reference genes in different developmental stages of porcine oocytes and IVF embryos. Relative abundance of the reference genes *GAPDH* (A), *B2M* (B), *H2A* (C) and *ACTB* (D), in porcine oocytes and early IVF embryos. Data are presented as the mean ± standard deviation. *, *P* < 0.05; **, *P* < 0.01 between groups, as indicated.

## Data Availability

The datasets generated and/or analysed during the current study are available in the GSA repository (https://ngdc.cncb.ac.cn/gsub/submit/gsa/list), and the assigned accession number of the submission is: CRA006174, detailed accession numbers: MII_1 (CRX404164), MII_2 (CRX404165), MII_3 (CRX404166), IVV_2C_1 (CRX335537), IVV_2C_2 (CRX335538), IVV_2C_3 (CRX335539), IVV_4C_1 (CRX335540), IVV_4C _2 (CRX335541), IVV_4C_3 (CRX335542), IVV_8C_1 (CRX335543), IVV _8C_2 (CRX335544), IVV_8C_3 (CRX335545), IVV_Bla_1 (CRX335546), IVV_Bla_2 (CRX335547), IVV_Bla_3 (CRX335548), SCNT_2C1 (CRX335549), SCNT_2C2 (CRX335550), SCNT_2C3 (CRX335551), SCNT_4C1 (CRX335552), SCNT_4C2 (CRX335553), SCNT_4C3 (CRX335554), SCNT_8C1 (CRX335555), SCNT_8C2 (CRX335556), SCNT_8C3 (CRX335557). Some or all data, models, or code generated or used during the study are available from the corresponding author upon request.

## Abbreviations

SCNT: Somatic Cell Nuclear Transfer
IVV: In Vivo developed
MZT: Mother-to-zygote transition
ZGA: Zygote genome activation
TF: Transcription factor
DEGs: Differentially expressed genes
PCP: Parallel coordinate plot
IVF: In vitro fertilization
PZM3: Porcine zygote medium 3
GO: Gene ontology
IF: Immunofluorescence
SPSS: Statistics production for service solution
PPI: Protein-protein interaction
TUNEL: Terminal deoxynucleotidyl transferase dUTP nick-end labeling
