# Supplementary figures and images for: Profiling the transcriptomic signatures and identifying the patterns of zygotic genome activation – a comparative analysis between early porcine embryos and their counterparts in other three mammalian species
Source: BMC Genomics. 2022 Nov 24;23:772. doi: 10.1186/s12864-022-09015-4 (PMC9700911; doi:10.1186/s12864-022-09015-4)

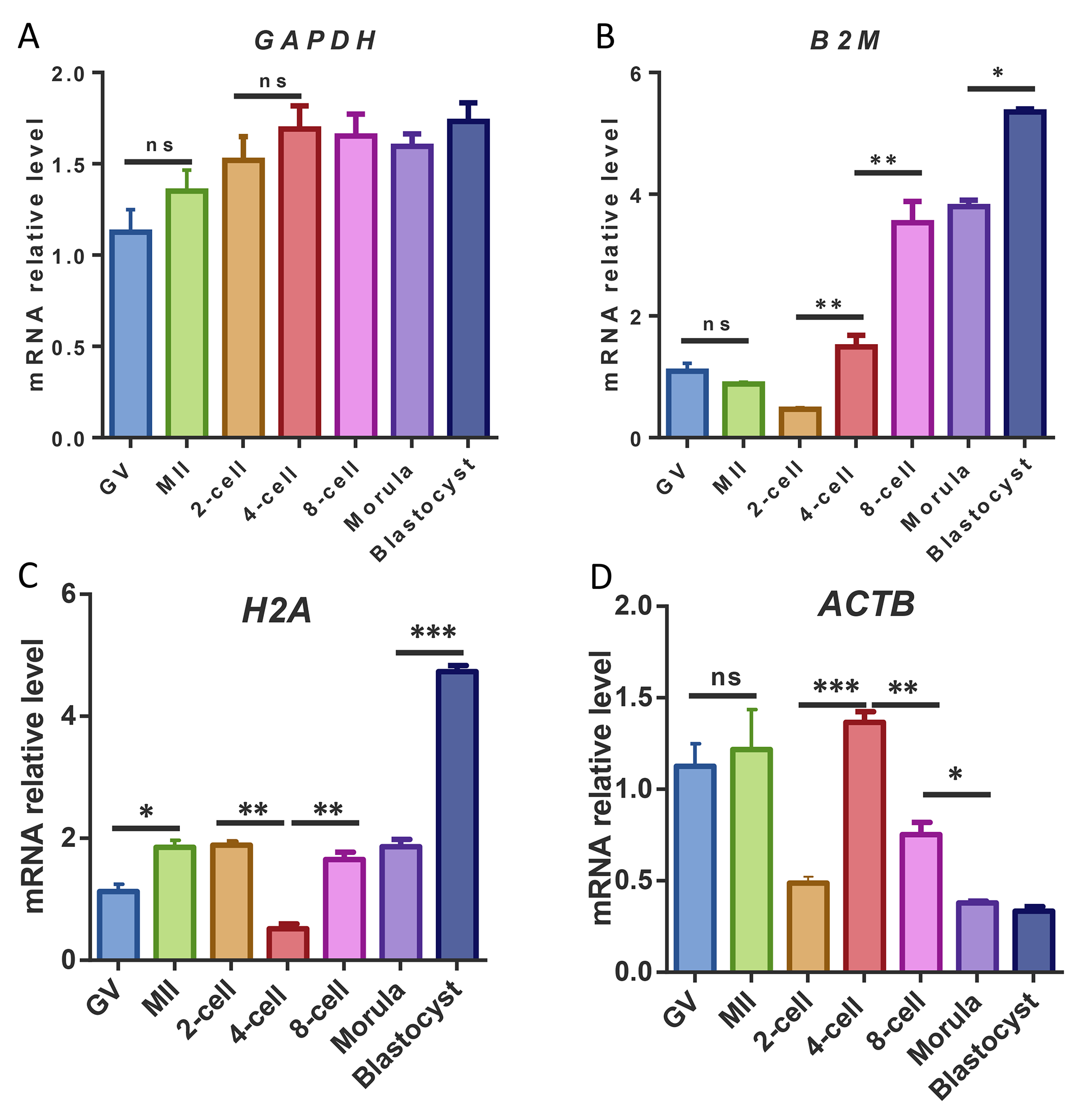

Supplement: Supplementary file 1 — Additional file 1: Supplementary Fig. 1. Expression patterns of reference genes in different developmental stages of porcine oocytes and IVF embryos. Relative abundance of the reference genes GAPDH (A), B2M (B), H2A (C) and ACTB (D), in porcine oocytes and early IVF embryos. Data are presented as the mean ± standard deviation. *, P < 0.05; **, P < 0.01 between groups, as indicated. [file 12864_2022_9015_MOESM1_ESM.tif]
